# Supplementary material for: Are carbon emissions trading and green financial instruments synergistic? -Comprehensive quantitative research based on content analysis
Source: PLoS One. 2024 Mar 7;19(3):e0298601. doi: 10.1371/journal.pone.0298601 (PMC10919601; doi:10.1371/journal.pone.0298601)
Supplement: S1 Data — (ZIP) [file pone.0298601.s001.zip › Data/地级市Z_F工作报告.zip!/║┌┴·╜¡/╦τ╗»╩╨/└┤╫╘╣1⁄2╓┌║┼í╛┬φ┐╦╩2╛▌═°í┐_2012.docx]

各位代表:

我代表市人民政府，向大会报告工作，请予审议，并请政协委员提出意见。

一、过去五年及2011年政府工作回顾

过去五年，我们认真贯彻省委、省政府“八大经济区”、“十大工程”发展战略，以及市委一系列决策部署，解放思想，抢抓机遇，攻坚克难，奋力拼搏，全市经济社会发展取得重要成就。这五年，是经济实力显著增强的五年。与2006年相比，预计2011年全市地区生产总值达到880亿元，增长1.2倍,同比增长15%；全口径财政收入98亿元，增长3倍，同比增长32%；一般预算收入42亿元，增长3倍，同比增长47%，其中市本级全口径财政收入22亿元，增长3.6倍，一般预算收入6亿元，增长6.3倍，同比分别增长20%和62%；规上工业增加值135亿元，增长2.3倍，同比增长28%；固定资产投资407亿元，增长4.5倍，同比增长35%；城镇居民人均可支配收入11100元，农民人均纯收入7719元，分别增长1倍和1.6倍，同比分别增长17 %和20.8 %。肇东连续三年位居全省十强县之首、并作为全省唯一全国百强县位次前移到85位，安达稳居全省十强县前三位。是上项目调结构成果丰硕的五年。累计对上争取新农村建设、现代农业、扶贫开发、良种补贴、土地整理等农业项目资金28亿元，是前五年的3倍。粮食总产突破300亿斤，增长近1倍。全市引进投资超3000万元工业项目600个，超亿元工业项目341个，项目总数是前五年的2.5倍，工业固定资产投资增长5.5倍，规上工业企业总资产达到408亿元。专业物流、旅游、金融等现代服务业项目增多。三次产业结构由34:23：43调到35：27:38。是城市面貌发生可喜变化的五年。城市建设规模总量翻番。铺装改造城镇道路493万平方米，排水管网586公里，分别是前五年的2.34倍、2.67倍。新增大中型城市广场绿地23处，绿化覆盖率达到30%，增长11个百分点。新增集中供热面积1928万平方米。城市房地产开发面积2589万平方米，是前五年的5.6倍。市本级拉开中等规模城市框架。绥棱、望奎、肇东、安达等地生态城、滨水城建设取得突破。是发展改善惠及民生最多的五年。各种直接惠农补贴超过70亿元；完成棚户区拆迁面积800多万平方米，改造泥草房21.43万户，35万多户城乡居民住上新房，增加财产性收入65.6亿元；补发了国有企业拖欠职工工资；累计新增就业30.1万人；5.4万名农村60周岁以上老人领取养老金。累计发放企业职工养老金68亿元，兑现医疗保险待遇9亿元，发放低保救助金30多亿元。学校、医院、社区、文化体育场馆、“三供两治”、公路客运等公共设施建设全面加强。城乡群众生活水平明显提高。是可持续发展能力不断提升的五年。投资7.49亿元如期完成松花江流域污染防治规划项目；投资11.7亿元，新上10个污水处理厂，城市上百年污水直排历史即将结束；投资7.8亿元，新上54个工业减排项目。建成20个自然保护区，总面积2580平方公里，占国土面积的7.4%。认真落实省委公路建设三年决战部署，投资62.3亿元，建设农村公路16473公里，建设总量连续五年全省第一。按照双业主制要求，确保了绥伊、绥北两条高速公路如期建成通车。这些，都为可持续发展提供了强力支撑。五年来，全市其他各项事业也都取得明显成绩。

2011年是承上启下的一年，是大发展、大跨越、亮点纷呈的一年。在市委的坚强领导下，我们认真贯彻落实2.28常委扩大会议以来市委一系列新的战略、思路、机制、措施，全市经济社会发展取得重大突破，各项主要经济指标均实现超常规增长。不仅圆满结束了本届政府任期，更为顺利推进“十二五”规划和今后五年的政府工作奠定了坚实基础。

一是同步强力推进招商引资和园区建设，产业项目建设成效前所未有。全市开工建设投资超3000万元产业项目368个，总投资800亿元。其中，亿元以上项目203个；异地项目112个，总投资200亿元，拉动乡财收入达到16亿元，同比增长70%；工业项目341个，完成工业固定资产投资249亿元，同比增长63.7%；有160个产业项目建成试生产，占开工项目的43%。安达太平庄40万吨芳构化项目当年建设、当年投产，运行仅40天就纳税1500万元。深入实施异地经济战略，放大资源共享优势，重点打造了市本级、肇东、安达、兰西四个产业项目集中区，以及安达万宝山化工产业园等一批专业园区。望奎、海伦、青冈等园区功能进一步完善。全市园区基础建设总投入14.7亿元，是去年的2.9倍，园区影响力、承载力、集聚力跨越提升。产业项目和园区建设有力拉动了工业经济增长，为绥化经济社会快速可持续发展积蓄了后劲。全市规上工业总产值434亿元，同比增长 36.1%，利税53亿元，同比增长32%。

二是突出抓好标准和载体建设，现代化大农业和新农村建设迈出坚实步伐。突出粮食高产创建。实现了粮食总量8连增，总产达305.8亿斤，纯增36.7亿斤，占全省增量三分之一。突出示范引带。着力打造千公里科技示范带。在示范带上共落实标准化示范点188个，完成大垄整地76万亩。突出体制机制创新。新组建规范的农民专业合作社404家，旱田规模经营面积496万亩，农业产业化龙头企业发展到128家。突出基础保障能力。投入农机化建设资金10亿多元，新组建千万元以上农机合作社56个，新增大型农机具1499台（套）。新增大型喷灌设备1207台（套），新增灌溉面积50万亩。新增农村饮水安全工程690处，惠及31万人。十个县（市）区全被列为全国小型农田水利建设重点县。突出科技推广应用和标准化生产。推广先进实用种养技术15项，新增设施棚室2.8万亩，新增规模养殖场（区）296个，新增水稻催芽基地18处，供苗能力150万亩。完成秋整地1976万亩，其中深松整地1012万亩。突出新农村建设。总结推广了新农村建设五种模式，整乡整村推进取得重大突破。全市村镇建设总投资达47.6亿元，30个重点小城镇建设全面铺开，完成了27个新农村示范村整村建设，撤并自然屯46个。投资3.7亿元，新修农村公路1017公里。全市新农村建设进入了由政府出资建到多元投资建、由点状分散建到全市统筹建、由侧重建房屋到综合建环境的可持续发展阶段。

三是统筹推进建城、管城、兴城，城镇化建设实现重大转变。立足建设中等规模城市群，发展城市型经济，大力完善提高城市功能。全市城市基础设施建设投入15.6亿元。新建改造城市道路117万平方米、排水管网139公里；新增公园绿地237公顷；新增集中供热563万平方米；安装改造路灯3285盏。争取国家和省道路交通和危桥改造资金10亿元，进一步改善了城市和城际之间的路网条件。市本级和9个市县都启动了中等规模城市规划，海伦、兰西、青冈、安达、肇东、望奎等地都启动了新城区建设。全市城市新增建设用地640公顷，房地产开发1009万平方米。市本级通过开展市容美化行动，城市面貌秩序显著改善。共清理违建违占5000多处；新建城市道路13条；投入4500万元，提升康庄路等主要街路绿化美化层次，新增公园绿地113公顷。坚持公益为先，不惜减少财政收入4000万元，把原定用于房地产开发的老体育场等三处地块改建公共绿地；改造更新了中心市场等四个农贸市场；整顿规范了房地产开发建设秩序，为中心城市健康发展奠定了坚实基础；新上了森林植物园、西湖公园等重要生态项目。各地都加强了城市管理，管理效能明显提高。望奎县被评为全国文明城市。建城、管城、兴城正在实现着城市让生活更美好，环境让城市更宜居，发展让人民更幸福，文化让绥化更和谐。

四是优化提升现代服务业，商贸经济活力显著增强。总投资48亿元，开工建设79个现代服务业项目。全年实现社会消费品零售总额294亿元，同比增长17.5%；进出口贸易额1.27亿美元，同比增长22.6%；实际利用外资1.3亿美元，同比增长20%；新增银行业支行机构9家、小额贷款公司16家，机构覆盖率全省第一，公司开业率全省第三。物流业规模扩大、纳税能力增强，旅游收入上升。

五是致力扩大富民惠民成果，民生和社会事业进入崭新层次。全市财政投入社会保障就业和医疗卫生资金31.3亿元。城镇新增就业6.5万人。新增企业养老保险1.97万人。138万农民参加了新型农村养老保险。农林牧渔四场1.94万职工纳入企业职工养老保险。城镇职工、居民医保和新农合政策范围内最高报销比例达到75%、65%、70%。城乡低保人数达51.69万人，实现了应保尽保。农村五保对象集中供养率达到60%以上。全市9万多名地方关破企业退休人员纳入城镇职工基本医疗保险。三年医改阶段性目标任务全部完成。市本级第一医院、妇幼保健院晋升三级甲等医院。在市本级建成了我市第一座全省一流的儿童福利院。全市1459名孤残儿童的养育、康复、教育、医疗得到有力保障。投入21.5亿元，超额完成校安工程、公办幼儿园和标准化学校建设任务，提高了义务教育经费保障标准。教育改革顺利推进，教学管理全面加强。全市开工建设保障房44503套，改造泥草房5.2万户。提高了市本级职工住房公积金等五项标准，年增资近7000万元。

六是加强社会管理创新，形成了政治安定、社会稳定的良好局面。社会治安打防控和综合治理体系进一步完善，有力震慑了违法犯罪，刑事和治安案件发案率同步下降。切实维护群众合法权益，民情在线畅达民意、解决民难的作用日益明显，稳控物价取得一定成效，追缴拖欠农民工工资和保障金7350万元。深入开展信访百日专项治理和领导干部大接访活动，全市信访矛盾排查率、调处率、办结率均提高到95%以上。加大教育、食品、药品、交通、建筑、石化等重点行业和重点领域安全隐患整治力度，有效遏制了重特大事故的发生。加强政府廉政勤政建设，强化依法行政，有力保障和促进了和谐绥化建设。

监察、审计、科技、人口计生、气象、防震减灾、统计、档案、民族宗教、妇女儿童、外事侨务、民兵预备役、双拥优抚、国防人防、残联、地方志和中省直部门等单位的各项工作都取得明显成绩。

五年来，特别是2011年，在落实市委一系列重大改革创新举措的实践中，我们更加深刻地体会到，只要牢固树立科学发展观，立足发展根本，坚持从实际出发，持之以恒转变发展方式，就一定能够保持经济社会更好更快发展；只要不断深化体制机制创新、科技创新，就一定能够保持发展的生机和活力；只要把富民和强市有机结合起来，以民为本、民生优先，就一定能够持续提高人民生活水平，保持社会和谐稳定；只要求实务实，真抓实干，看准的事情咬住不放，一抓到底，就一定能够干大事、创大业，推动各项工作不断迈向新台阶。

各位代表！五年的变化令人振奋，发展成绩来之不易。这是省委、省政府和市委正确领导的结果；是人大、政协和社会各界朋友鼎力支持的结果；是历届班子奠定坚实基础的结果；是全市广大干部群众创新发展、苦干实干、共同奋进的结果。为此，我代表本届政府表示衷心的感谢！并致以最崇高的敬意！

全面总结过去五年工作，我们也认识到，我市经济社会发展中还存在着一些亟待解决的矛盾和问题：一是人均经济总量小，经济发展相对滞后；二是产业结构、财政收入结构不合理，农业大而不强，工业支撑能力弱，三产层次较低，特别是财政收入中不稳定因素占一定比重；三是市本级立市工业项目少，城市承载能力不强，改造老城区、开发新城区，建设区域中心城市任务繁重。四是教育、卫生等民生事业发展水平不高与满足广大群众现实需求的矛盾还比较突出；五是社会管理亟需加强。这些矛盾和问题，我们一定高度重视，认真解决。

二、未来五年经济社会发展的主要任务

未来五年，是顺利完成“十二五”规划、推进经济社会大发展、大跨越的关键转折期，是经济发展方式实现重大转变的战略机遇期，更是考验我们践行发展是第一要务、民生是第一宗旨、稳定是第一责任的重大挑战期。客观分析形势，尽管国际金融危机的影响短期内很难消除，全球性通胀压力和经济下行的风险仍在，经济发展的不确定因素增多，一些负面因素不可避免影响和传导到我市。但总体上看，绥化借势发展、优势发展、强势发展、创新发展的历史机遇前所未有。国际国内经济转型、产业转移、生产要素流动加快，以及绥化自身农牧资源、劳动力资源和地处龙江中心地区的优势，更有利于我们引进更多项目和资金；转变发展方式、促进经济平稳较快发展，是国家今后一个时期宏观调控的主基调，更有利于我们抢夺后发优势，抓紧优化项目结构，推动经济发展加快升级转型；国家和省持续加大对“三农”、民生、城乡一体化发展的政策支持力度，更有利于我们向上争取政策支持。特别是适应“八大经济区”、“十大工程”战略要求，市委从绥化巨大的优势潜力和必须加快发展的市情出发，成功创新实施了异地经济、都市圈经济、园区经济、城市型经济、乡镇村经济等战略举措，不仅指明了绥化大发展、大跨越的战略方向、推进重点和科学途径，更高度统一了全市上下的认识和行动，坚定了广大干部群众图强奋进的信心和决心。这是我们展现新作为、谋求新跨越的最难得机遇、最有利条件、最根本动力。邓小平曾说：“我们什么错误都可以犯，就是不能犯丧失机遇的错误”。我们一定要倍加珍惜难得的发展机遇，倍加珍惜人气旺盛、精诚团结的良好局面，倍加珍惜来之不易的蓬勃发展势头。未来五年，政府工作总的要求是：以科学发展观为统领，深入贯彻中央、省委、省政府的总体战略部署，特别是市第三次党代会精神，加快建设全省一流的工业经济和产业园区、一流的现代化大农业、一流的城市型经济和中等规模城市群、一流的社会主义新农村、一流的民生和社会事业，强力推进园区经济、都市圈经济、异地经济、乡镇村经济、城市型城市群经济五大战略的落实，更高水平推进经济社会大发展大跨越，民生水平大改善大提升，各项工作大提档大晋位，加快实现中心崛起、富民强市目标。

在全面完成“十二五”规划的基础上，未来五年,全市经济总量和财政收入等主要经济指标翻一番半以上，力争翻两番。地区生产总值达到2200亿元，年均增长20%以上；财政总收入220亿元，年均增长25%以上；工业增加值1000亿元，年均增长30%以上；固定资产投资1600亿元，年均增长30%以上；社会消费品零售总额700亿元，年均增20%以上；外贸进出口总额3.2亿美元，年均增长20%以上；城镇居民可支配收入和农民人均纯收入分别达到23000元和16000元，年均分别增长15%以上。

——建设全省一流的工业经济和产业园区，加快建成现代新兴工业重要集聚区。通过两轮的三年产业项目攻坚，“五大经济”战略调控和优势作用充分释放，布局合理、结构优化、分工明确、特色突出、集群发展的十大产业基本建成，绿色食品、高新化工、生物医药、纺织服装、新材料新能源等一批骨干产业集群迅速壮大。做到数量与质量并重、速度与效益同步，工业经济的主导地位基本确定。园区基础设施投入50亿元以上，固定资产投资1000亿元以上，新上扩建投资超3000万元产业项目2200个以上，每个园区都能打造出投资50亿元以上、销售收入100亿元以上的大企业、大集团。市本级、肇东、安达三个园区力争成为国家级开发区，其它八个县级园区进入省级开发区。金融、保险、物流等新兴服务业态与园区经济紧密融合。11个园区成为全市经济的增长极。工业总产值实现3000亿元，增加值占国内生产总值45%以上。

——建设全省一流的现代化大农业，承担起平原地区创建现代化大农业的重任。着眼农业产业化、农区工业化、农村城镇化、城乡一体化，积极推进集规模农业、合作农业、装备农业、设施农业、科技农业、生态农业于一体的现代农业多种有效实现形式。农业基础建设显著加强，旱田有效灌溉面积达到800万亩，大型农机合作社发展到300个以上，农民专业合作社发展到4200 家，规模连片种植面积1500万亩以上，粮食总产突破360亿斤。标准化畜牧场（区）发展到800个，畜牧业占农业比重50%以上。农产品精深加工比重达到80%以上。

——建设全省一流的城市型经济和中等规模城市群，加速实现从大农业、大农村向大工业、大城市跨越。规划城市、建设城市、管理城市、经营城市、发展城市统筹推进。中心区位优势、都市圈叠加优势、县域自主发展优势充分发挥。市本级、肇东、安达、海伦、兰西、绥棱、青冈七个中等规模城市，望奎、庆安、明水三个区域中心城市框架基本拉开。城市工业发达、商贸繁荣，具有开放、规模、集聚、扩散效应的城市型经济比重达到75%以上。城市建成区面积达到230平方公里，增加50平方公里，新增建筑总面积3000万平方米，城市绿化覆盖率达到43%，人均住房面积达到30平方米，城镇登记失业率控制在4.5%以内，城市教育、医疗、文化和基础设施等城市功能更加完善，每年新增城市人口8万人以上，城镇化率达到46 %以上。

——建设全省一流的社会主义新农村，从根本上改变农村面貌和农民生产生活条件。按照建好县城、建好重点小城镇、建好中心村、适度保留农业作业点和畜牧养殖基地的发展思路，建设重点示范小城镇30个以上，五星级示范村300个以上，撤并自然屯500个，农村中心服务社区实现全覆盖。发展产业化，住房现代化，道路网络化，造林网格化，饮水安全化。村有城的环境，乡有城的文明。乡镇村产业税收实现100亿元，占财政收入比重40%以上。以七个中等规模城、三个区域中心城为核心，以新兴小城镇为纽带，以中心村和中心社区为基点，城乡一体、布局合理、产业发展、生活宽裕、环境宜居的现代化新农村格局初步形成。

——建设全省一流的民生和社会事业，使绥化人民生活的更幸福、更富裕、更有尊严。城乡就业渠道更宽，社会保障能力更强，基本公共服务更好，社会文明程度更高。累计新增城镇就业15万人以上；城乡养老保险、低保救济实现全覆盖，三项医疗保险参保率达到97%；教育、医疗条件更加满足群众需求，文化活动丰富多彩，精神生活健康向上。政治安定，社会稳定。城镇居民年人均增收2000元以上，农民年人均增收1500元以上。

实现上述任务目标，必须突出把握好以下几点：一是必须突出贯彻落实市委一系列新的发展战略。实施“五大经济”、“五个一流”等一系列创新举措，是市委贯彻落实科学发展观的重要成果，是指导全市经济社会科学发展、更好更快发展的总体战略，必须毫不动摇地抓好贯彻落实，让每一个经济都能迸发出无限活力，让每一个一流都能催生出不竭动力，加快实现跨越赶超。二是必须突出产业发展、扩张经济总量。大上项目、上大项目，快上项目、上好项目，靠项目成就产业，靠产业拉动增长，做活存量，做大增量。三是必须突出优化结构、转变发展方式。做强传统优势产业，做大战略新兴产业；重点发展城市经济，加快发展乡镇村经济，大力发展生态、创税型经济。调强产业结构，调优经济结构。提高产业投资占固定资产投资比重，税收占财政收入比重，财政收入占GDP比重。四是必须突出创新发展。聚焦重点，主攻难点，深化体制、机制创新和科技创新，推动经济社会发展进入创新驱动轨道。五是必须突出改善发展民生。以富民为先，无论困难多大，保障民生的力度不能减；无论压力多重，改善民生的投入必须留足，强力推进民生事业晋档升位。六是必须突出和谐稳定。加强社会建设，创新社会管理，努力建设平安绥化、幸福绥化、和谐绥化。

三、全面做好2012年政府工作

2012年，是新一届政府的开局之年，做好今年的工作至关重要。我们要全面贯彻中央、省委经济工作会议和市三次党代会精神，从绥化实际出发，紧紧把握未来五年“强化五大经济、实现五个一流”战略重点，突出科学发展主题、转变发展方式主线、跨越发展主基调，以产业项目决战年、城市建设管理年、民生好事实事改善提升年为载体，抢抓机遇，真抓实干，优化结构，壮大总量，全面经营，全程管理，高起点开局、高标准起步、高速度跨越，强力推进各项重点工作实现新突破。

主要预期目标是：国内生产总值增长20%以上；规上工业增加值增长30%以上；固定资产投资增长32%以上；社会消费品零售总额增长20%以上，进出口贸易额增长20%以上，实际利用外资增长15%以上；财政总收入和一般预算收入分别增长25%以上；城镇人均可支配收入和农民人均纯收入分别增长15%以上；万元GDP综合能耗下降3.2%，主要减排指标达到规定要求。着力做好十个方面重点工作。

（一）深入推进产业项目决战年活动，在工业经济和园区建设上实现新突破

全力抓好两次集中签约、三次集中开工，项目总量在去年基础上实现翻番，工业质量大幅提高。强力推进招商引资。围绕十大产业，重点上固定资产投资超3000万元项目，突出上超亿元项目，优先上财税贡献型项目，积极上高新技术项目，坚决不上污染项目。节约土地资源，万平方米土地投资强度达到2000万元以上。全市11个园区都要至少上2个投资超5亿元项目、1个投资超10亿元项目，并力争引进投资超50亿元和100亿元的重量级项目。全年新建续建固定资产投资超3000万元项目700个以上、超亿元项目增长30%以上，实际完成投资增长50%，开工项目建成率达到50%。实现决战决胜。提高园区建设水平。进一步完善重点产业集中区和园区的规划设计及产业定位，超前做好规划环评。全面加强园区基础建设，加快提升市本级、肇东、安达、兰西、海伦等重点产业集中区的集聚能力。其他县区的园区也要提档升级。加强园区管理，理顺管理队伍和机构设置，建立决策咨询机构，向重点企业派驻服务人员。高度重视在园区内引进金融、保险、信息、物流等现代服务机构，打造优良的园区服务环境。提高工业经济运行质量。强化科技支撑，大力提升创新发展能力。支持企业技术改造和建立研发中心，努力使新产品生产、研发、储备紧密衔接，让科技真正成为撬动工业发展的有力杠杆。支持重点企业增强拉动能力，保证工业经济提速增效。加大节能减排力度，全年万元工业增加值综合能耗下降6%。

（二）统筹城乡发展，在推进现代化大农业和新农村建设上实现新突破

继续大力推进现代农业和新农村建设。毫不放松抓好粮食生产。确保粮食安全是我们的政治责任，必须突出抓好。要以建好千公里科技示范带、1000万亩粮食高产创建核心区、1000万亩辐射区、800万亩带动区为载体，深入实施粮食产能巩固提高工程，力争粮食产量达到320亿斤。优化种植结构，玉米、水稻、大豆面积调整到2000万亩、500万亩、100万亩。争取更多的项目和资金，广泛吸纳社会资金，切实加强以大水利、大农机、大科技为重点的农业基础建设。加快阁山水库立项审批。全市新增旱田有效灌溉面积100万亩。以建好农民合作社为核心，以解决生产关系适应生产力为前提，加快推进土地有序流转、连片种植。全市新建规范的农民专业合作社150家，旱田规模经营面积达到650万亩。千方百计增加农民收入。让农民尽快富起来，是我们各级政府的政治任务，必须全力抓好。靠调优种植结构增收。向高产、优质、高效上调。绿色食品种植面积达到1600万亩，棚室蔬菜等特色作物发展到200万亩；靠加快发展畜牧业实现过腹增收。新建畜牧示范园区10个，奶牛、生猪、肉牛、禽类等养殖数量增长9%以上，达到1.5亿头（只）；靠鼓励农民进入市场，搞活流通增收。规范提升10个农产品批发市场、165个集贸市场；靠大力发展劳务经济，实现内转外输增收。同时推进旅游观光农业发展，多渠道、多层次增收。大力推进农业产业化经营。这是城乡一体化的中轴，必须努力抓好。大上农产品精深加工项目，深入实施寒地黑土品牌战略，做大做强更多的龙头企业，打造绿色“大粮仓经济”、“大厨房经济”，进一步拉动农业增效、农民增收、财政增税。继续推进新农村建设。做好撤屯、减村、并镇、建城规划，积极推广新农村建设“五种模式”。每种模式每个县至少建设一个示范村，大县强县要力争达到2个以上，新增整村建设示范村60个以上。加快小城镇建设。以创建“三优”文明城市为载体，加快建设好位于交通干线主轴、近郊和列入全省百镇试点的城镇。抓好新一轮连片扶贫开发，提高新农村整体建设水平。

（三）放大城市功能，在推进城市型经济和中等规模城市群建设上实现新突破

深入开展城市建设管理年活动。坚持辟建新城区、改造旧城区、建设产业园区三位一体，建管并重，把兴城作为重中之重。完善城市规划。按照园林式、生态型、现代化的总体定位，坚持城市规划与园区、新农村建设规划统筹考虑，重点围绕打造区域中心城、新型工业城、现代生态宜居城，高标准制定好中等规模城市群建设规划。市、县两级年内总体规划修编和小区的控详规划设计同步完成，增强规划的科学性和操作性。抓好城市建设管理。适应城市中长期发展需要，结合新老城区开发改造，加强城市给排水等地下管网、城市路网和绿色生态体系等基础建设，着力解决基础设施老化、广场绿地少和配套服务功能缺失问题。9县（市）新建改造给排水管网100公里以上、道路100条以上，新增绿地380公顷，绿化覆盖率达到35%，新改扩建城市广场10个以上，主次街路全部实现立体亮化。合理划分社区范围，完成社区用房建设。解决好集中供热，力争消灭“小锅炉”。同时，配套跟进教育、文化、金融、社区医疗等公共服务设施。加强城市管理，推进城管向制度化、数字化、机械化、社会化、市场化转变。推进新城区建设。按照建设中等规模城市群的要求，合理规划配置城市优势资源，同步拉开新城区和产业园区框架，打造好重要节点，加大新城区建设的突破力度。重点推进安达、肇东、海伦、兰西、青冈、望奎等地的新区建设。全市新增房地产开发面积1000万平方米、建成区面积10平方公里。

（四）提升现代服务业，在推进商贸经济发展上实现新突破

优化结构，扩大总量，提高现代服务业在商贸经济中的比重。突出发展现代物流业。以市本级、肇东、安达、海伦、青冈等城市为主要节点，积极推进专业市场和物流工贸园区建设。发展壮大安达石化、海伦大豆、兰西亚麻、庆安水稻、肇东绿色食品等专业物流中心。加快重点专业市场升级改造。扶持第三方物流企业加快发展。大力发展金融服务业。支持银行业发展，引进、发展交通银行绥化分行和建行发起的肇东村镇银行等金融机构和组织10家以上，发展融资性担保公司10家以上。发挥好财政资金、国有资产的撬动引导作用，采取贴息、担保、以奖代补、扩大授信额度等措施，增加银行信贷规模，全市银行贷款余额300亿元以上。积极发展新兴服务业。抓住大上产业项目有利契机，积极发展信息、技术、外包、评估、咨询、专业培训等服务，打造特色化、专业化功能街区。适应扩大内需，大力发展文化、旅游、健身、养老、家政、社区服务，提升商贸、餐饮等传统服务业档次，扎实推进“万村千乡市场”工程，深入实施“农超对接”工程。主动参与全省旅游名镇建设，努力争取政策、资金支持。加强对外经贸合作。积极开拓国际市场，优化进出口结构，抓好园区跨境合作，新增有实绩直接进出口企业6家以上。

（五）突出惠民富民和社会管理创新，在推进民生和社会事业建设上实现新突破

大力开展改善提升民生的“好事实事工程”，重点抓好十个方面50件好事实事：

1、抓好富民增收。（1）大力发展特色农业和畜牧经济增加农民收入，力争人均纯增1500元以上。（2）强力发展产业项目增加全民就业和职工收入，城镇居民人均增收2000元以上。（3）搞活商贸流通增加全民收入。对400个农家店进行信息化改造。（4）促进创业带动就业，发放小额创业贷款3000万元。（5）鼓励支持中小企业加快发展，新增规模企业30户。全年新增城镇就业5万人以上。（6）加强劳动力就业培训，转移农村劳动力118万人，创收89亿元。

2、建好安居工程。（7）年内完成棚户区改造拆迁面积82万平方米，建设保障房18000套，其中廉租房5000套、公租房300套。（8）改造农村泥草房4.5万户。（9）鼓励支持符合条件的用户全部或部分购买廉租房产权。

3、加强社会保障和扶贫救助。（10）城镇企业养老参保人数达到26万人，新型农村社会养老保险实现全覆盖。（11）城镇居民医疗保险、新农合参保率分别达到96%、98%以上，继续提高医保待遇。（12）抓好失业、生育保险，推进工伤保险市级统筹。（13）提高城乡低保标准，城市月人均提高到265元，农村年人均提高到1350元。（14）建设县乡中心敬老院10所，五保村30个。城市社区居家养老达到70%以上。农村五保对象集中供养率达到65%。（15）完成扶贫开发整村推进40个，实现3万人脱贫。

4、强化城乡环境建设。（16）坚决拆除私搭滥建，治理规范牌匾广告，清理规范店外经营。（17）强化裸土治理，全面实现绿化硬化；结合新旧城区开发改造，建设完善垃圾中转站，增加卫生保洁设备，提高城市卫生管理水平。（18）治理农贸市场，改善经营环境。（19）抓好城市主要街道和楼群立体亮化。（20）增加市本级停车场、停车位，改善商业区交通环境。（21）大力发展城市公交。市本级增加开发区、东富工业园等公交线路，完成公交站点新建改造任务。（22）整顿规范出租车市场，严厉打击黑车，具备条件的安装出租车计价装置。（23）推进“三供两治”。加快推进城市二次供水集中改造，加大“一户一表”改造力度；新增饮水安全村屯500处，解决25万人的饮水安全问题；新增城市集中供热600万平方米，新增燃气管道用户8000户；市本级和9个县（市）污水处理厂全部投入正常运营；医疗垃圾集中无害化处理率达到95%；加强汽车尾气的监测和治理。（24）完善农村路网，新建通乡通村公路1000公里。

5、规范物业管理。（25）严把准入关，物业企业资质合格率达到100%。（26）物业严格按标准收费达到100%。（27）全面整顿物业企业不作为、乱作为，建立健全各项制度，规范管理达到100%。

6、优先发展教育。（28）全面推进教育绩效工资配套改革。着力解决教师结构性短缺。（29）搞好幼儿园建设，新改扩建公办幼儿园30所。（30）全面提高教育教学质量。学前教育三年普及率达到57%，高考二表以上进段率达到30%。办好职业教育，大力培养适用人才，中职招生9000人。（31）抓好标准化校舍建设，完成5.4万平方米“校安工程”。（32）整顿规范校车，加强校园周边环境治理，严格管理校内食堂和“小饭桌”卫生防疫，确保学生高兴上学，安全回家。（33）做好农村中小学布局规划，利用五年时间完成撤并任务。

7、提升文化惠民。（34）推进文化大发展大繁荣，全面提高公民的文化素质。建设绥棱黑陶、兰西民俗风情旅游等九个文化产业基地。（35）加快公共文化服务体系建设。新建农家书屋176个，新增文化广场300处、文化活动场所60处。（36）广泛开展群众性文体活动。推动文化、科技、卫生“三下乡”，发展广场文化，打造文化活动品牌。（37）开展好全民健身活动，全面提高竞技体育水平。建设全民健身工程100处以上。办好市直第四届运动会。

8、改善就医条件。（38）加大财政扶持力度，引进人才，更新设备，加快推进市本级第一医院进入全省先进行列。市本级人民医院医疗中心年内投入使用。（39）完善城乡医疗卫生服务体系，新改扩建乡镇卫生院3个、村标准化卫生所50个。（40）在政府举办的基层医疗卫生机构，严格执行药品零差价销售，减轻群众药费负担。（41）继续推进县级医院提档升级。以实施绩效工资改革为重点，加快公立医院改革。（42）实施人口计生“惠家工程”，严格兑现当年的独生子女奖励费，历史欠账补发20%以上。加强妇女儿童工作，落实好发展规划。

9、加强社区建设。（43）强化社区基础建设，社区服务用房300平方米以上达到65%，80%以上街道建成800平方米以上社区服务中心。农村社区覆盖率达到70%。（44）加强社区机构和队伍建设，建设社区综合服务站158个，城市每个社区专兼职工作人员达到5—7人。（45）保障社区经费，社区办公经费和工作人员的工资补贴纳入财政预算。（46）推进政府公共服务职能和工作重心下沉，建好城市社区和乡镇信息化综合服务平台。

10、抓好社会稳定。（47）加强信访工作，进一步完善责任体系和工作机制，全市信访量减少20%，矛盾排查率、调处率、办结率均达到95%以上。（48）加强社会治安综合治理，以高压态势严厉打击违法犯罪，降低案件发案率，综合破案率比上年提高10%以上。（49）强化以食品药品安全、生产安全为重点的公共安全监管，严厉打击假冒伪劣和商业欺诈行为，坚决遏制重特大安全事故发生。（50）积极做好物价稳控工作，用好价调基金，对困难群体价格补贴达到1000万元以上。严厉打击各种价格违法行为。

去民之患，如除腹心之疾。我们一定要克服困难、解决问题，坚持民生财政、民生预算，努力为群众办更多的好事、实事。

（六）放大共享优势，在推进异地经济发展上实现新突破

大力发展异地经济。全年异地落地项目达到200个以上。科学统筹规划。紧密结合生态功能区划分，实行异地经济集聚区差异化发展。在产业重点、功能定位上各有侧重，推进优势项目向优势地区集中，使全市的优势产业形成布局合理、分工明确、各具特色的集聚板块。打造异地经济专区。在市本级、肇东、安达、兰西、海伦等五个重点产业集中区内，重点建设好异地经济专区，其他县的园区也可作为异地经济发展区，促进全市范围内的项目科学摆布、集群发展。在市本级、肇东和安达开发区启动建设总部经济服务园，承接总部经济项目。完善利益共享机制。财政、税务、工商等部门，创新管理机制，促进异地经济发展。

（七）激活乡镇村行政单元，在推进乡镇村经济发展上实现新突破

转变乡镇村经济发展方式，构筑以现代农业为基础、异地工贸产业为主导的新型乡镇村经济。全市乡镇财政收入增长50%。加大乡镇村招商引资力度。充分发挥乡镇村行政单元多、干部队伍大、人脉资源广的优势，采取重奖重用，定任务、落责任、统一政策等激励措施，强力推动乡镇村主动招商、全员招商，实现乡镇村招商全面突破。推进乡镇村经济集约发展。乡镇村引进的超亿元项目，重点落户到全市十一个县级以上园区。在重点乡镇打造承接中小项目的产业园区，形成各具特色的集聚区、产业带、企业群。加强乡镇村经济管理。对招商引资形成的乡村两级财力纳入市、县两级财政监管范围，实行预算控制、审批使用、收支两路、集中财力，项目收益用于公益事业和产业滚动发展，以钱生钱，增强乡村两级“造血”功能。

（八）挖掘放大优势潜力，在推进都市圈经济发展上实现新突破

突出资源共享、市场共用、产业共兴、发展共赢，推动都市圈经济加快发展。建设多层次的都市圈经济。充分利用毗邻哈大齐的地缘优势、交通便利优势、三大城市人才技术信息资金等要素荟萃集聚的优势和辐射带动功能强大的优势，以及我们具有的盐碱地、农牧资源、劳动力、电力、生态等优势，大力发展与三大城市一样类型的同体型经济，以及为其产业服务配套的协作型经济、吃配型经济，跻身其产业分工；大力发展以新技术、新兴产业为主的高端领军型经济，抢占发展制高点；大力发展以食品、化工、医药等为主导的优势特色型经济，扩大在全省同类产业中的比重；大力发展都市圈内的服务型经济、城郊型经济，努力在物流配送、绿色食品供应、劳动力培训输出、生态旅游等方面，服务全省、走向全国。打造都市圈经济集聚区。在抓好11个重点园区的同时，面向哈大等重点城市接壤地带，重点打造好肇东五站高端产业园区，安达万宝山石化工业区、卧里屯商贸特区，兰西哈北新城等八个都市圈经济集聚区。优化都市圈经济发展环境。全力促进大开放、大合作、大开发，与哈大齐等城市建立更加紧密的合作关系。加快城际之间、产业集聚区与城市之间的快捷通道建设,助推都市圈经济加快发展。

（九）抓好全面经营、全程管理，在推进经营增收、管理增效上实现新突破

加强全面经营、全程管理，是理念，是责任，更是政府工作的重要任务。在坚定不移扩大投资增量促发展的同时，进一步在投入讲回报、资金讲节约、成本讲预算、经营讲实效、管理讲规范上下功夫。用精心、恒心、狠心，经营管理好我们的一切优势资产和资源，促进经济收入和社会效益的显著提升，加快发展方式转变。重点经营管理好政府资产。整合现有一切可利用的资产，通过建立政府控股的城投公司或担保公司等方式，盘活存量资产，增加地方财政收入。加快做大做强市本级城投公司。加强政府投资项目管理，实行工程预决算全过程财政评审和审计监督，杜绝跑冒滴漏。严格各级财政预算管理，严格税收征管，真正做到收入进财政、支出按预算。重点经营好城市。把城镇作为资源和产业来精心经营、精细管理。打造最佳的经营环境，最佳的经营区域，形成新的经济圈，凸显新的增长点。以城养城，以城兴城，以城惠民。帮助重点企业搞好资本运营。扶持企业联营联合，上市融资，做大做强，发展总部经济，实现裂变式扩张，滚动式发展。重点挖掘和经营最具潜力、活力及优势的资源项目。注重在经营机遇、政策、会展、品牌、教育、卫生等诸多资源上下功夫，在经营全过程中的每一个环节、细节上，用心思、动脑筋、展智慧，真正把全面经营、全程管理这篇文章做大、做精、做活，实现全面的经营增收、管理增效。

（十）围绕建设区域中心强市，在推进市本级加快发展上实现新突破

做大做强市本级，壮大综合实力，切实把市本级变成全市政治、经济、文化中心。加强财源建设。注重争取外援，更加注重自主创收，尽快实现向自有财力保运转、促发展，对上争取扩大再发展上转变。靠经营所有的优势资产资源增收，靠管理服务好现有企业和新上项目扩大增量。树立过紧日子思想，努力开源节流。2012年，市本级一般预算收入增长25%以上。加大招商引资力度。深入贯彻落实市直机关招商引资工作实施意见。各级领导率先行动，机关干部全员发动，激励问责，严格考核，使招商引资成为一种常态，成为衡量领导干部实绩的主要标准，切实在招商引资数量和质量上实现大突破。年内确保新上亿元以上项目28个。发展壮大经济开发区。推进基础设施建设，完成园区道路20公里、绿化面积20万平方米；建设3万平方米的开发区综合服务中心，力争年内投入使用。采取突破性措施，确保项目用地。加快推进园区项目建设，确保维维豆奶、中豆食品、白象方便面、石头造纸等8个项目年内竣工投产，确保远景新能源、大北农科技园等20个以上超亿元项目年内开工建设。全面加强开发区管理，完善开发区机构职能设置，招聘10名以上大学生充实开发区人才队伍。从开发区和市直部门选调精干力量组成6个包扶组，帮助企业协调解决技改、资金、环境等实际问题，促进企业增产增效、扩大再生产。年内开发区工业增加值、利税同步增长30%以上。坚持市区一体发展，统一政策，整合资源，统筹抓好市区两级项目摆放。全力提升中心城市建设管理水平。按照中心大城市框架，高水平做好区域中心城市总体规划修编。建成科研路、西湖公园、森林植物园，加速推进新城区开发；抓好16项棚户区改造续建工程，完成31万平方米拆迁任务，完成棚改和商品房开发150万平方米。年内完成19项排水工程、13项道路工程，同步高标准完成主城区绿化和立体亮化工程。完成第二水厂、垃圾处理厂改造、污水处理厂二期建设，积极推进红星水库招商，力争完成大唐集中供热项目。加强城市管理，深入开展城市环境卫生、交通运管、市场秩序、私搭滥建等专项治理工作，巩固扩大成果，建立长效机制。

同时，其他各项工作，也要全面贯彻落实国家、省和市委的要求部署，完善思路，创新措施，取得新的更大突破。

四、切实加强政府自身建设

做好2012年和未来五年的各项工作，使命光荣，任务艰巨。必须全面加强政府自身建设，进一步提高政府的落实力、执行力、创新力、公信力。

1、加强学习，提高能力。把学习作为政治责任和第一需要，紧密围绕发展方式大转折、产业项目大攻坚、五个经济大举措、五个一流中心崛起富民强市大目标，深入开展学习型政府班子、学习型领导干部、学习型公务员队伍建设活动，学习理论与创新实践相结合，博采众长、完善自我，以学立德、以学增智，在理论中把握方向，在实践中总结规律，切实把学习的成果转化为实践能力，培养造就一批发展项目、服务企业的专才，全面经营、全程管理的通才，敢于负责、善于解决急、难、险、新问题的能才，进而把思想行动高度统一到市委决策部署和人大决议、决定上来。

2、规范行为，依法行政。主动自觉接受人大、政协的依法监督和民主监督，认真办理人大代表的议案和政协委员的提案。完善与民主党派、工商联、无党派人士的信息沟通机制，畅通新闻媒体和社会各界监督渠道。健全听证、论证、集体决定、结果公示相结合的行政决策机制。严格执行重大决策程序，提高政府决策的规范性和严密性。发挥市长电话、市长信箱、民情在线、群众来信来访了解民情、反映民意的作用，保障广大人民群众的知情权、参与权、表达权、监督权，广泛凝聚各方力量和智慧，建设绥化、发展绥化。

3、创新落实，提高效能。落实要力度，关键在干部，提效是核心，创新生动力。要把落实的基点建立在对发展的信心、创优的恒心、工作的热心、事业的忠心、群众的诚心上，创新实践，勇于突破，真抓实干，求质重效。突出转变职能，常规工作向突破转，行业工作向中心转，局部工作向全局转，用新思维研究新情况，用新制度解决新问题，用新举措推进新发展。强化效能监察，完善绩效评估，继续推行重大决策部署专项督察制度，完善重点工作和重大项目考核制度，以制度说话，按目标考核。狠抓务实求实落实，把政令畅通作为严肃的政治纪律和工作纪律，不走样、不跑粗，说到做到、做就做好，坚决消除中梗阻。年底前，每个市直政府部门都要确保完成和超额完成规定的招商引资任务。

4、转变作风，改善环境。牢固树立群众观念和群众立场，强化公仆意识，坚持深入基层、深入实际，问政于民、问需于民、问计于民，努力做到感情上与群众“心连心”，工作上与群众“面对面”，办事上与群众“实打实”。年内每个政府部门都要拿出一篇有份量的解决民生、经济发展、社会管理等方面问题的调查报告。继续做好政府领导、部门领导信访接待日活动。大力倡导明察暗访、现场办公等方式解决问题。改善发展环境。深入推进政务公开和政府信息公开向基层延伸；深化行政审批制度改革，推行并联审批，减少环节，提高效率；清理行政事业性收费，规范行政执法，从体制机制上解决多头执法、交叉执法问题。加强诚信体系建设。积极推进政务诚信、商务诚信、社会诚信，以诚信赢得相信，以行动和实效取信于民。

5、从严治政，廉洁从政。廉政才能公正。要严守廉洁自律的警戒线、党纪国法的高压线，加强惩治和预防腐败体系建设。建立完善自身督查、任期审计、行政监察相结合的内部监督体系，斩断公权为部门、为个人寻租途径。加大对土地出让、政府采购、工程招投标、国有资产处置等重点领域的行政监察和审计监督力度，严肃查处各类违纪违法案件。在巩固离任审计的基础上，加强任期审计，今年任期审计面达到20%。积极推进事业单位改革，加强人事编制管理，杜绝自聘人员。倡导艰苦奋斗，反对铺张浪费，建设节约政府。今年，政府经费不突破财政预算。

各位代表！以市委二届七次全委（扩大）会议和三次党代会为标志，绥化的发展又站在了一个新的起点上。让我们在市委的坚强领导下，以科学发展观为统领，凝神聚力谋发展、心无旁骛创大业、砥砺奋发勇争先，为更高水平实现“五个一流”、中心崛起、富民强市目标而努力奋斗！
